# PD-L1 Blockade Differentially Impacts Regulatory T Cells from HIV-Infected Individuals Depending on Plasma Viremia

**DOI:** 10.1371/journal.ppat.1005270

**Published:** 2015-12-03

**Authors:** Cristina Peligero, Jordi Argilaguet, Roberto Güerri-Fernandez, Berta Torres, Carmen Ligero, Pilar Colomer, Montserrat Plana, Hernando Knobel, Felipe García, Andreas Meyerhans

**Affiliations:** 1 Infection Biology Laboratory, Department of Experimental and Health Sciences, Universitat Pompeu Fabra, Barcelona, Spain; 2 Infectious Diseases Unit, Hospital del Mar, Universitat Autònoma de Barcelona, Barcelona, Spain; 3 Infectious Diseases Unit, Hospital Clínic, Barcelona, Spain; 4 Retrovirology and Viral Immunopathology Laboratory, AIDS Research Group, IDIBAPS, Hospital Clínic, University of Barcelona, Barcelona, Spain; 5 Institució Catalana de Recerca i Estudis Avançats (ICREA), Barcelona, Spain; Emory University, UNITED STATES

## Abstract

Blocking the PD-1/PD-L1 pathway has emerged as a potential therapy to restore impaired immune responses in human immunodeficiency virus (HIV)-infected individuals. Most reports have studied the impact of the PD-L1 blockade on effector cells and neglected possible effects on regulatory T cells (Treg cells), which play an essential role in balancing immunopathology and antiviral effector responses. The aim of this study was to define the consequences of *ex vivo* PD-L1 blockade on Treg cells from HIV-infected individuals. We observed that HIV infection led to an increase in PD-1+ and PD-L1+ Treg cells. This upregulation correlated with disease progression and decreased under antiretroviral treatment. Treg cells from viremic individuals had a particularly high PD-1 expression and impaired proliferative capacity in comparison with Treg cells from individuals under antiretroviral treatment. PD-L1 blockade restored the proliferative capacity of Treg cells from viremic individuals but had no effect on its suppressive capacity. Moreover, it increased the viral production in cell cultures from viremic individuals. This increase in viral production correlated with an increase in Treg cell percentage and a reduction in the CD4/Treg and CD8/Treg cell ratios. In contrast to the effect of the PD-L1 blockade on Treg cells from viremic individuals, we did not observe a significant effect on the proliferative capacity of Treg cells from individuals in whom viremia was controlled (either spontaneously or by antiretroviral treatment). However, PD-L1 blockade resulted in an increased proliferative capacity of HIV-specific-CD8 T cells in all subjects. Taken together, our findings suggest that manipulating PD-L1 *in vivo* can be expected to influence the net gain of effector function depending on the subject’s plasma viremia.

## Author Summary

HIV infection causes a progressive impairment of effector immune responses, contributing to virus persistence. The restoration of these responses is essential to achieve a drug-free control over HIV. One strategy that could restore effector immune responses is the relief of the inhibitory signal displayed by the PD-1/PD-L1 pathway on effector cells. However, the PD-1/PD-L1 pathway also plays a role in the biology of regulatory T cells, which in turn suppress effector responses. Here we show that *ex vivo* PD-L1 blockade on peripheral blood mononuclear cells from HIV-infected individuals differentially increases the proliferative capacity of regulatory- and effector- T cells depending on the subject’s plasma viremia. Our results suggest that PD-L1 blockade will skew the effector-to-regulatory T cell ratio in favour of effector cells only in patients in whom viremia is controlled. In patients with uncontrolled viremia, PD-L1 blockade will not favour effector- T cells over regulatory- T cells, and might also boost virus reactivation. Our findings support the rationale to combine a PD-L1 blockade with antiretroviral treatment to restore effector responses in HIV-infected individuals.

## Introduction

Inhibiting programmed cell death 1 (PD-1) signalling has a potential therapeutic value for treating cancers and persistent viral infections (reviewed in [1–5]). PD-1 is a co-inhibitory receptor that plays a major role in exhaustion, a dysfunctional state of effector cells caused by antigen persistence [6]. Exhausted T cells present defects in effector function including impaired proliferation, cytotoxic capacity and cytokine production. These defects can be partially restored by blocking the interaction between PD-1 and its ligand programmed death ligand-1 (PD-L1), which notably reduces viral loads in several animal infection models [7–10]. This observation has also been extended to important persistent human infections such as the human immunodeficiency virus (HIV) infection, both *in vitro* [11–14] and *in vivo* in HIV-infected humanized mice [15,16]. Since the HIV load is directly correlated with disease progression [17], an augmentation of antiviral immune responses by blocking the PD-1/PD-L1 pathway might help to control viral replication and slow down pathogenesis. Furthermore, it may facilitate clearance of latently infected cells, and thus may represent a promising strategy to reach a functional cure of HIV infection [18,19].

PD-1 and PD-L1 are expressed on several cell types including regulatory T cells (Treg cells) [20]. Treg cells are a suppressive T cell subset mediating self-tolerance and immune homeostasis (reviewed in [21,22]). During HIV-infection, Treg cells have both, beneficial and detrimental roles (reviewed in [23–25]). For example, Treg cells control excessive immune activation that limits immunopathology and the availability of HIV target cells. On the contrary, Treg cells contribute to the destruction of the lymphatic tissue architecture, and inhibit HIV-specific immune responses promoting virus persistence. Therefore, any therapeutic alteration of Treg cell numbers and function may directly influence the balance between immunopathology and viral control.

PD-L1 blockade therapy in HIV-infected individuals is expected to affect their Treg cells. Indeed, several roles of the PD-1/PD-L1 pathway are already described for this cell subset. For example, PD-1/PD-L1 pathway is essential in the induction of Treg cells in the periphery [26–28] and the maintenance of their suppressive capacity [28–33]. PD-1 is also described as a negative regulator of Treg cells in hepatitis C virus infection [34]. Likewise, *in vivo* blockade of PD-L1 increased the numbers of Treg cells in the Friend virus mice model [35]. In the context of a HIV infection, PD-1 was found up-regulated in Treg cells compared with healthy controls [36–38]. Nonetheless, as most reports have focused on effector cells, possible effects from PD-L1 blockade on Treg cells have been neglected. In light of the upcoming therapeutic trials blocking PD-L1 in HIV-infected patients, it is important to understand its consequences for Treg cells, as they are essential players balancing immunopathology and antiviral effector responses.

The purpose of this report was to investigate the impact of *ex vivo* PD-L1 blockade on Treg cells from HIV-infected individuals. We found that PD-L1 blockade had no effect on Treg cells’ suppressive capacity. However, PD-L1 blockade increased the proliferative capacity of Treg cells from viremic individuals but had no significant effect on Treg cells from individuals that control viremia. Interestingly, we found that PD-L1 blockade in peripheral blood mononuclear cells (PBMC) from viremic individuals increased virus production. This increase was related with increased Treg cell frequencies, suggesting that the inhibitory function of Treg cells may play a role in virus expansion upon PD-L1 blockade. In contrast to the differential effect from PD-L1 blockade on Treg cell proliferation, we observed an increase in the proliferation of effector T cells in all groups of HIV–infected individuals. Therefore, manipulating PD-L1 *in vivo* is expected to influence the net gain of effector function depending on the subject’s plasma viremia.

## Results

### PD-1 and PD-L1 are increased on Treg cells from HIV-infected individuals

**Fig 1:**
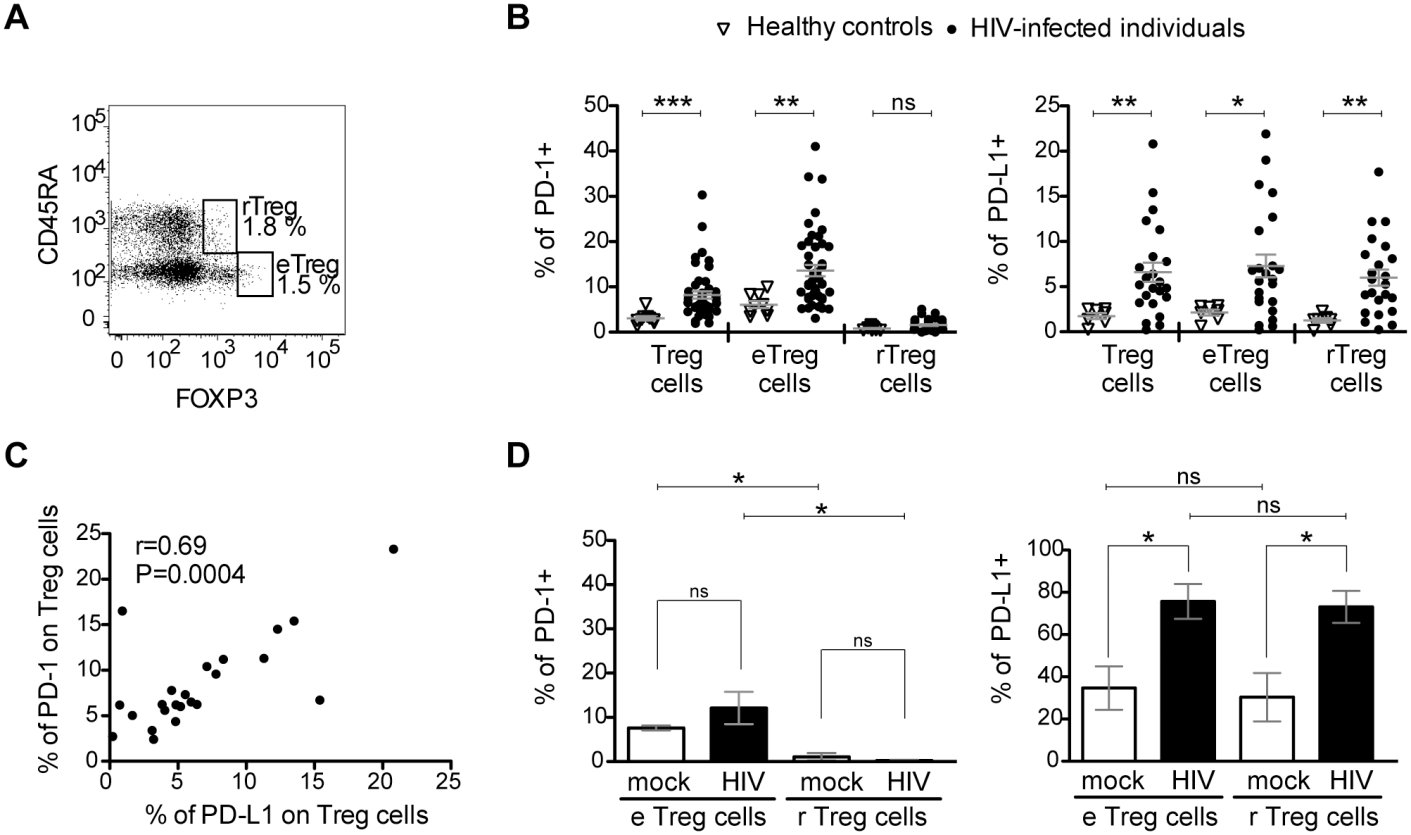
The percentage of PD-1 and PD-L1 expressing Treg cells is increased in HIV-infected individuals. (A) A representative flow cytometry dot plot from an HIV-infected individual with <500 CD4/μL and >2000 viral RNA copies/mL blood showing the gating of effector Treg cells (eTreg: CD4+CD45RA-FOXP3^hi^) and resting Treg cells (rTreg: CD4+CD45RA+FOXP3^lo^) from a previously gated CD4 T cell population. Numbers indicate percentages of each population. (B) Percentages of PD-1+ (left panel) and PD-L1+ (right panel) Treg (including eTreg and rTreg), eTreg and rTreg cell populations in HIV-infected individuals (black circles) and healthy, uninfected controls (empty triangles). The mean ± SEM (standard error of the mean) is shown. Significant differences were determined by a Mann-Whitney U test and indicated by asterisks (*p <0.05; **p <0.01; ***p <0.001; ns: non significant). (C) Correlation between PD-1 expression and PD-L1 expression on Treg cells from HIV-infected individuals. The Spearman’s rank correlation coefficient (r) and the p value (P) are indicated. (D) Expression of PD-1 (left panel) and PD-L1 (right panel) in healthy control’s PBMC exposed to HIV-1 Bal (black bars) at 0.3 multiplicity of infection, compared with mock control (white bars). Bars represent the mean ± SEM (standard error of the mean) from 3 independent experiments with 7 different donors. Significant differences were determined by Wilcoxon matched pairs test and indicated by asterisk (*p <0.05). ns, non significant.

Previous studies have shown that (i) PD-1 is overexpressed on CD4- and CD8- T cells in several persistent infections and cancers, and (ii) that this overexpression plays a key role in the exhausted phenotype of these cells (reviewed in [39]). To first evaluate the expression of PD-1 and its ligand PD-L1 on Treg cells from HIV-infected individuals, we used the gating strategy of Miyara and colleagues [40]. It distinguishes between effector Treg cells (eTreg, CD4+CD45RA-FOXP3^hi^) and resting Treg cells (rTreg, CD4+CD45RA+FOXP3^lo^) (Figs 1, S1 and S2). The advantage over the traditional Treg cell characterization by CD4+CD25^hi^CD127^lo^FOXP3+ is that conventional CD4 T cells with an up-regulated CD25 and FOXP3 expression due to the generalized, infection-related immune activation are excluded from the analysis [41]. PBMC from HIV-infected individuals and healthy controls were isolated, stained with fluorescence-labelled antibodies and characterized by flow cytometry. A significantly higher percentage of PD-1+ Treg cells was observed for HIV-infected individuals (8.2% ± 0.8 SEM) compared with controls (3.0% ± 0.4 SEM) (Fig 1B). This difference in PD-1 expression was due to PD-1 on effector Treg cells (13.6% ± 1.2 SEM) since very little PD-1 was expressed on resting Treg cells (1.5% ± 0.2 SEM). These observations are concordant with previous data [36–38] and fit to the current understanding of PD-1 upregulation induced by T cell stimulation [42]. We also found a higher percentage of PD-L1+ Treg cells for HIV-infected individuals (6.59% ± 1.1 SEM) compared with controls (1.75% ± 0.33 SEM) (Fig 1B). However, in contrast to the expression pattern of PD-1, an increased percentage of PD-L1+ cells was observed for both, effector and resting Treg cells. Furthermore, the expression of PD-1 and PD-L1 on Treg cells from HIV-infected individuals correlated positively (Fig 1C).

The differential distribution of PD-1 and PD-L1 on resting and effector Treg cells from HIV-infected individuals and healthy controls suggested that the virus itself could induce PD-L1 upregulation on Treg cells. To test this hypothesis, PBMC from healthy controls were isolated and exposed to HIV-1Bal containing supernatants without additional stimuli or additional interleukin-2. As controls, we used supernatants from non-infected PBMC that have been cultured under similar conditions as the virus-exposed cells. While culture supernatants from non-infected PBMC increased the frequency of PD-L1+ effector and resting Treg cells, virus exposure dramatically augmented this effect in a dose-dependent manner (Figs 1D and S3A). In contrast, virus exposure had no effect on PD-1 expression (Fig 1D).

To evaluate whether PD-L1 upregulation occurred without infection, we cultured PBMC with infectious HIV-1Bal in the presence or absence of the HIV entry inhibitor T20. PD-L1 upregulation on Treg cells occurred after virus exposure even in the presence of T20 (S3B Fig). These data are in line with previous studies that demonstrate a PD-L1 upregulation upon HIV exposure in different cell populations including monocytes, macrophages, dendritic cells, neutrophils and CCR5+T cells [43–46]. In addition, we observed that the HIV-envelope protein gp120 induced PD-L1 upregulation in Treg cells providing an extra mechanistic insight into how HIV can induce PD-L1 (S3C Fig). When taken together, our results show an upregulation of PD-1 and PD-L1 on Treg cells of HIV-infected individuals that may be mediated by different routes and suggest that the Treg cell compartment is likely to be influenced by immunotherapy targeting the PD-1/PD-L1 pathway.

### PD-1 expression on Treg cells is associated with disease progression

**Fig 2:**
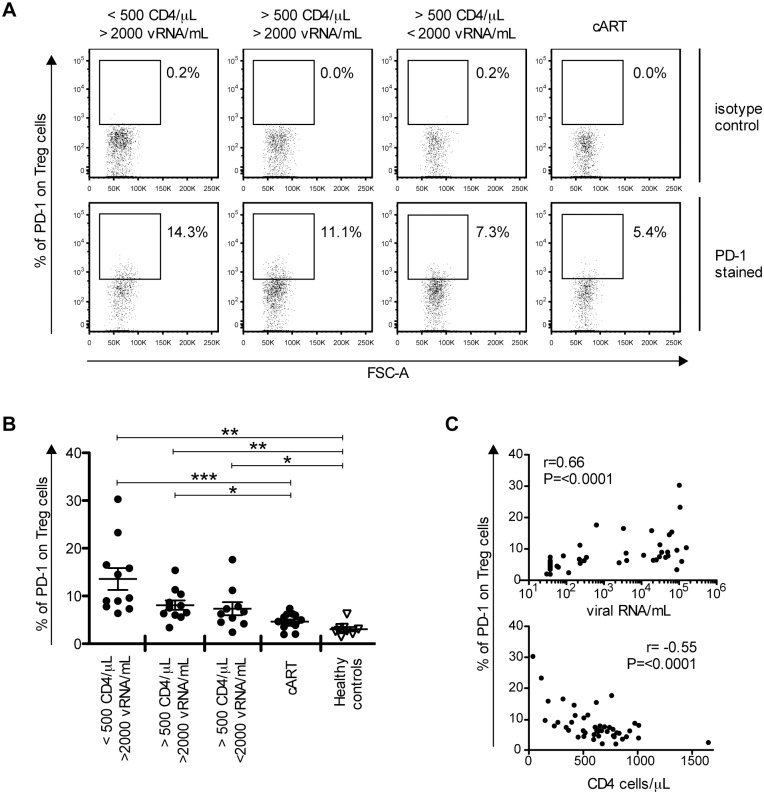
The frequency of PD-1-expressing Treg cells correlates with markers of disease progression. (A) Representative flow cytometry dot plots showing PD-1 gating on Treg cells (including eTreg and rTreg). One HIV-infected individual from each study group is displayed. Numbers indicate the percentage of PD-1+ Treg cells in PD-1 stained samples (down) compared with isotype control antibodies (up). (B) PD-1 expression on Treg cells from different HIV-infected study groups (black circles) and healthy controls (empty triangles) are shown as indicated. Each dot represents the result from one individual. The mean ± SEM (standard error of the mean) is shown. Significant differences were determined by a Mann-Whitney U test, corrected for multiple comparisons using Bonferroni method, and indicated by asterisks (*p <0.05; **p <0.01; ***p <0.001). (C) Correlation of PD-1 expression on Treg cells from HIV-infected individuals with viral loads (up) and CD4 T cell counts (down), respectively. Spearman’s rank correlation coefficients (r) and p values (P) are indicated.

PD-1 expression on CD4- and CD8- T cells correlates with HIV disease progression [11,13,47]. To test whether the same is true for Treg cells, we analysed PD-1 expression on these cells from HIV-infected individuals categorized into 4 groups according to CD4 T cell counts and viral load (S2 Table). The highest percentage of PD-1-expressing Treg cells was found in the HIV study group with the lowest CD4 T cell counts and highest viral loads (Fig 2B). As many as 13.6% ± 2.3 SEM of Treg cells where PD-1+ in this group whereas only 4.7% ± 0.4 SEM of Treg cells where PD-1+ in the group of individuals under combination antiretroviral therapy (cART). PD-1 expression on Treg cells paralleled PD-1 on total CD4-T cells but differed from CD8-T cells (S4A Fig). Within viremic individuals, the percentage of PD-1+ CD8 T cells was high irrespective of the CD4 T cell counts, whereas the percentage of PD-1+ Treg and PD-1+ total CD4 T cells was higher in individuals with low CD4 T cell counts (<500 CD4/μL).

**Fig 3:**
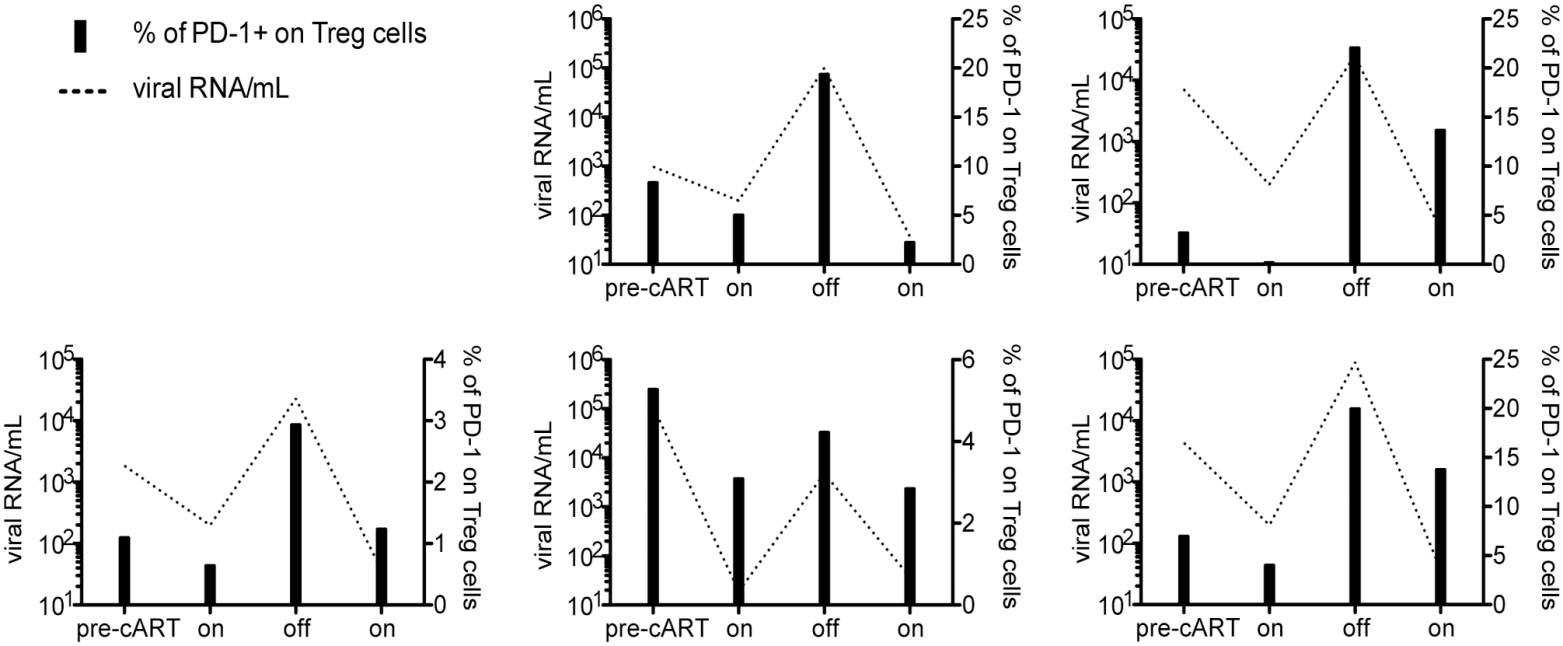
PD-1 expression on Treg cells follows HIV viremia. PD-1 expression on Treg cells (including eTreg and rTreg) (black bars) from 5 individuals followed longitudinally before and after antiretroviral treatment interruptions (pre-cART, on, off, and on, respectively). Each graph represents one individual. The dashed line represents the plasma viral load.

Consistently, PD-1 on Treg cells correlated positively with viral load and negatively with CD4 T cell counts (Fig 2C). This is concordant with previous observations made for CD4 T cells and CD8 T cells from HIV-infected patients [11,13,47] and reproduced here with individuals of our study groups (S4B Fig). To further substantiate the relation between PD-1 expression on Treg cells and antigen exposure, we followed 5 patients before and after antiretroviral treatment interruptions. Samples were collected from the same patient at 4 time-points, (1) before starting treatment, (2) during treatment, (3) upon interruption of treatment, and (4) after restarting treatment. As can be seen in Fig 3, the percentage of PD-1 expressing Treg cells followed viremia and cART reduced PD-1 expression on Treg cells.

**Fig 4:**
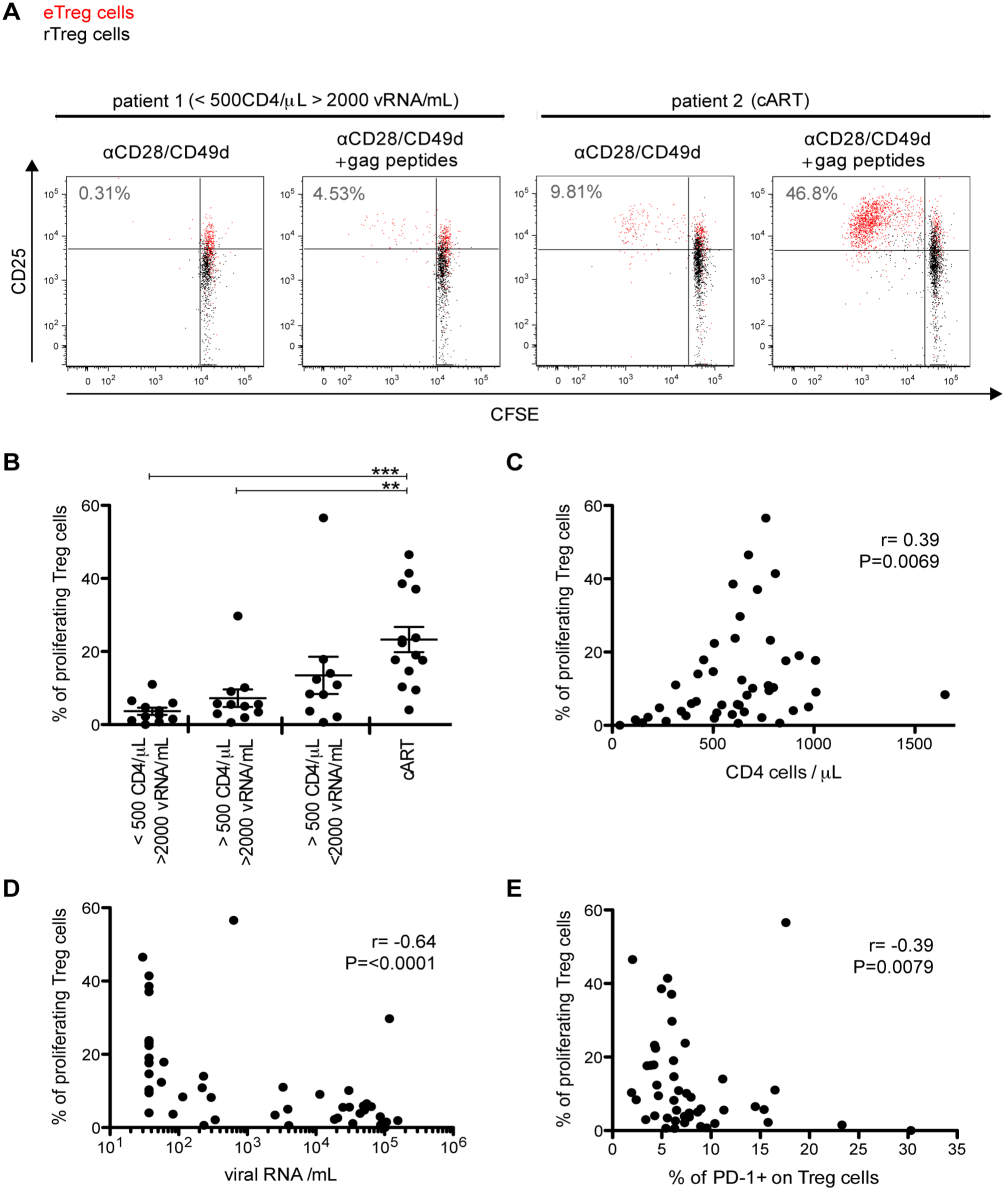
Treg cells from viremic individuals show impaired proliferative capacity that correlates with PD-1 expression. CFSE-labelled PBMC from HIV-infected individuals were stimulated with Gag peptides for 6 days. (A) Flow cytometry dot plots showing CFSE dilution of Treg cells (eTreg cells in red and rTreg cells in black). Numbers indicate the percentage of proliferating Treg cells (including eTreg and rTreg). (B) The percentages of proliferating Treg cells (including eTreg and rTreg) from different HIV-infected study groups are given as indicated. Each dot represents the result from one HIV-infected individual. The mean ± SEM (standard error of the mean) is shown. Significant differences were determined by a Mann-Whitney U test, corrected for multiple comparisons using Bonferroni method, and indicated by asterisks (**p <0.01; ***p <0.001). Panels C to E show correlations between the percentage of proliferating Treg cells and CD4 T cell counts (C), HIV viral load (D), and PD-1 expression on Treg cells before stimulation (E), respectively. Spearman’s rank correlation coefficients (r) and p values (P) are indicated.

PD-1 is a negative regulator of the proliferative capacity in effector T cells. To characterize the relationship between PD-1 expression and the proliferative capacity of Treg cells, PBMC from individuals of the different HIV study groups were labelled with Carboxyfluorescein succinimidyl ester (CFSE), stimulated with HIV Gag peptides and analysed for proliferation by CFSE dilution via flow cytometry. As shown in Fig 4B, the proliferative capacity of Treg cells was strikingly impaired in non-treated individuals. It correlated positively with CD4 T cell counts and negatively with viral loads (Fig 4C and 4D) and PD-1 expression on Treg cells prior to stimulation (Fig 4E). These observations parallel those reported for effector T cells [11] and suggest a negative role of PD-1 for Treg cell proliferation.

### PD-L1 blockade increases the proliferative capacity of Treg cells but not their suppressive capacity *per cell*

**Fig 5:**
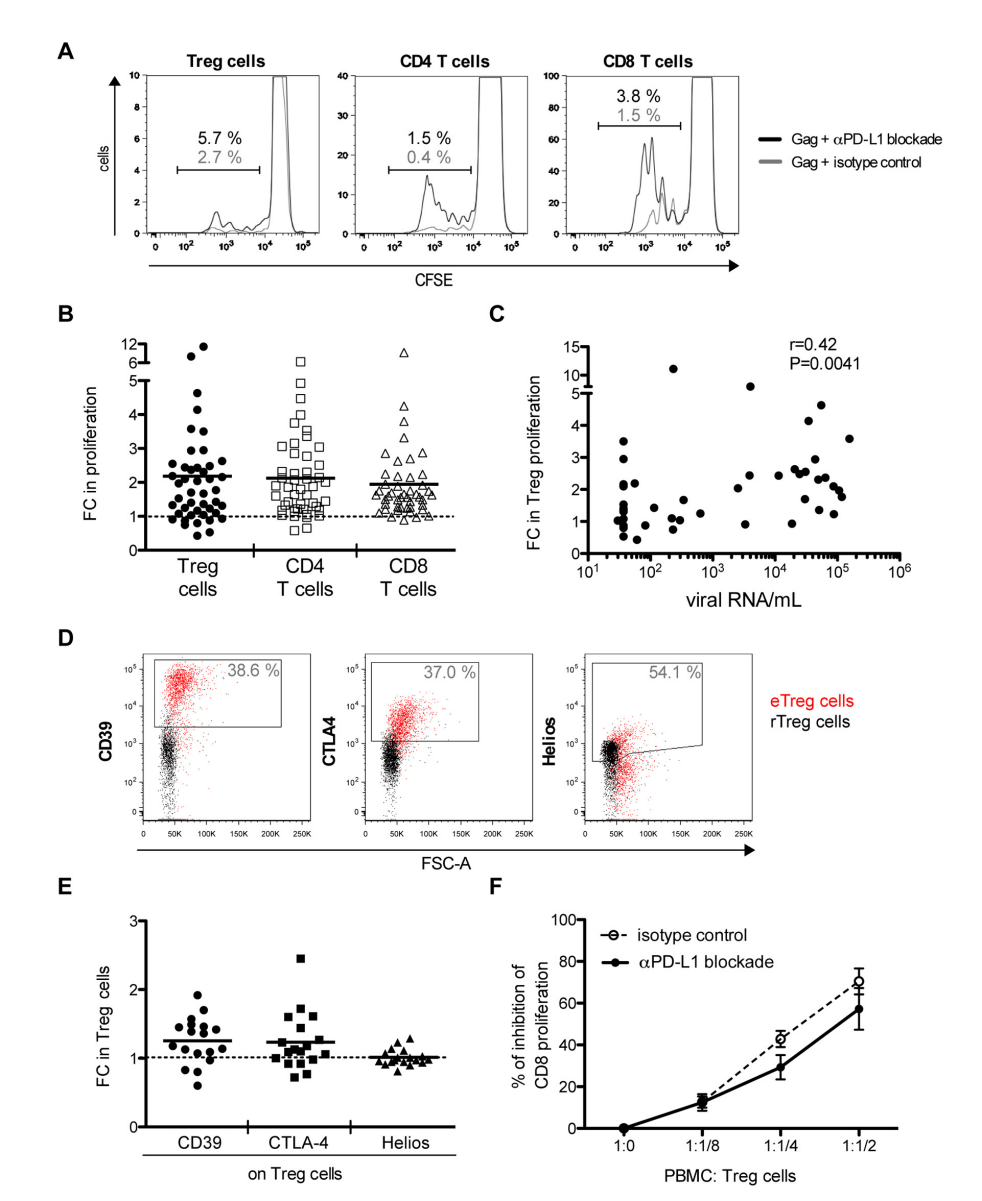
PD-L1 blockade increases Treg cell proliferation but not its suppressive capacity *per cell*. In panels A to E, PBMC from HIV-infected individuals were stimulated with Gag peptides for 6 days in the presence of a PD-L1 blocking antibody or an isotype control antibody. (A) Flow cytometry histograms from an HIV-infected individual with <500 CD4/μL and >2000 viral RNA copies/mL blood showing CFSE dilution on Treg (including eTreg and rTreg), CD4- and CD8- T cells in presence of PD-L1 blocking antibody (black line) or isotype control antibody (grey line). In panels B, C and E, each dot represents the result from one individual. FC (fold change) is calculated as the ratio between PD-L1 blockade conditions and isotype control conditions. The dashed line in panels B and E (FC = 1) indicates no change due to PD-L1 blockade. The mean is shown. (B) Fold changes in the proliferation of Treg (black circles), CD4 (empty squares) and CD8 (empty triangles) cell populations were determined by CFSE assay. (C) Correlation between the fold change in proliferating Treg cells and HIV viral load. (D) Representative flow cytometry dot plots showing gating of CD39, CTLA4 and Helios on Treg cells for one individual (eTreg cells in red and rTreg cells in black). Numbers indicate the frequencies of CD39, CTLA4 or Helios expressing Treg cells (including eTreg and rTreg). (E) Fold change in the frequencies of CD39, CTLA4 or Helios expressing Treg cells. (F) PBMC from HIV-infected individuals were stimulated with Gag peptides in the presence of a PD-L1 blocking antibody or an isotype control antibody, after 6 days Treg cells were isolated and co-cultured with CFSE-labelled-PBMC at different PBMC-to-Treg cell ratios. The percentage of inhibition of CD8 proliferation after anti-CD3/anti-CD28 T cell stimulation is shown as a function of different PBMC-to-Treg cell ratios. Black dots connected with a solid line correspond to suppression assays performed with Treg cells from PD-L1 blockade conditions whereas empty dots connected with a dashed line correspond to suppression assays performed with Treg cells from isotype control conditions. Mean and SEM from 4 independent experiments are indicated.

To analyse the impact of a PD-L1 blockade on the proliferative capacity of Treg cells from HIV-infected individuals, CFSE-labelled PBMC were cultured in the presence of HIV Gag peptides and PD-L1 blocking antibody or an isotype control antibody. Cell proliferation was quantified by CFSE dilution via flow cytometry. A significant gain on the proliferative capacity of Treg cells, as well as that of effector CD4- and CD8- T cells, is shown in Fig 5 as fold change in proliferation relative to the isotype antibody control stimulations (p <0.0001). PD-L1 blockade led to a roughly 2 fold mean increase in the percentage of proliferating Treg cells, comparable to that of effector CD4- and CD8- T cells (Fig 5B) (p = 0.668). The range of responses was broad. The increase in Treg cell proliferation positively correlated with the viral load of the analysed individuals (Fig 5C).

To further analyse the functional consequences that a PD-L1 blockade may have on Treg cell function after an antigenic stimulation, we analysed the increase of Treg cells expressing effector molecules such as CD39 and CTLA4 as well as their suppressive capacity. Upon PD-L1 blockade a slight but significant increase in the frequency of CD39- and CTLA4- expressing Treg cells was observed relative to the control condition (p = 0.026 and 0.039, respectively) as well as to the fold change in the percentage of Helios-expressing Treg cells (p = 0.027) (Figs 5E and S6A). The latter is a transcription factor suggested to identify thymic Treg cells, and used as a control. As expected, the frequency of Helios-expressing Treg cells did not increase upon PD-L1 blockade (p = 0.862). To test the capacity of expanded Treg cells to suppress CD8 T cell proliferation, Treg cells were isolated from PBMCs after a 6-day-culture in the presence of PD-L1 blocking antibody or an isotype control antibody, and co-cultured with CFSE-labelled PBMCs in the presence of anti-CD3/anti-CD28 and interleukin-2. Proliferation of CD8 T cells was quantified by analysing CFSE profiles by flow cytometry. A dose-dependent inhibition of CD8 T cell proliferation was observed that was not significantly different from that of isolated Treg cells expanded under control conditions (Fig 5F). Although the *in vitro* study of the suppressive capacity of Treg cells might not always be predictive of *in vivo* function, the presented data suggest that the relief of the PD-1/PD-L1 interaction during expansion does not alter the suppressive capacity of Treg cells on a *per cell* basis.

### The proliferative capacity of Treg cells and CD8 T cells from HIV-infected individuals is differentially restored by PD-L1 blockade and depends on the plasma viremia of the host

**Fig 6:**
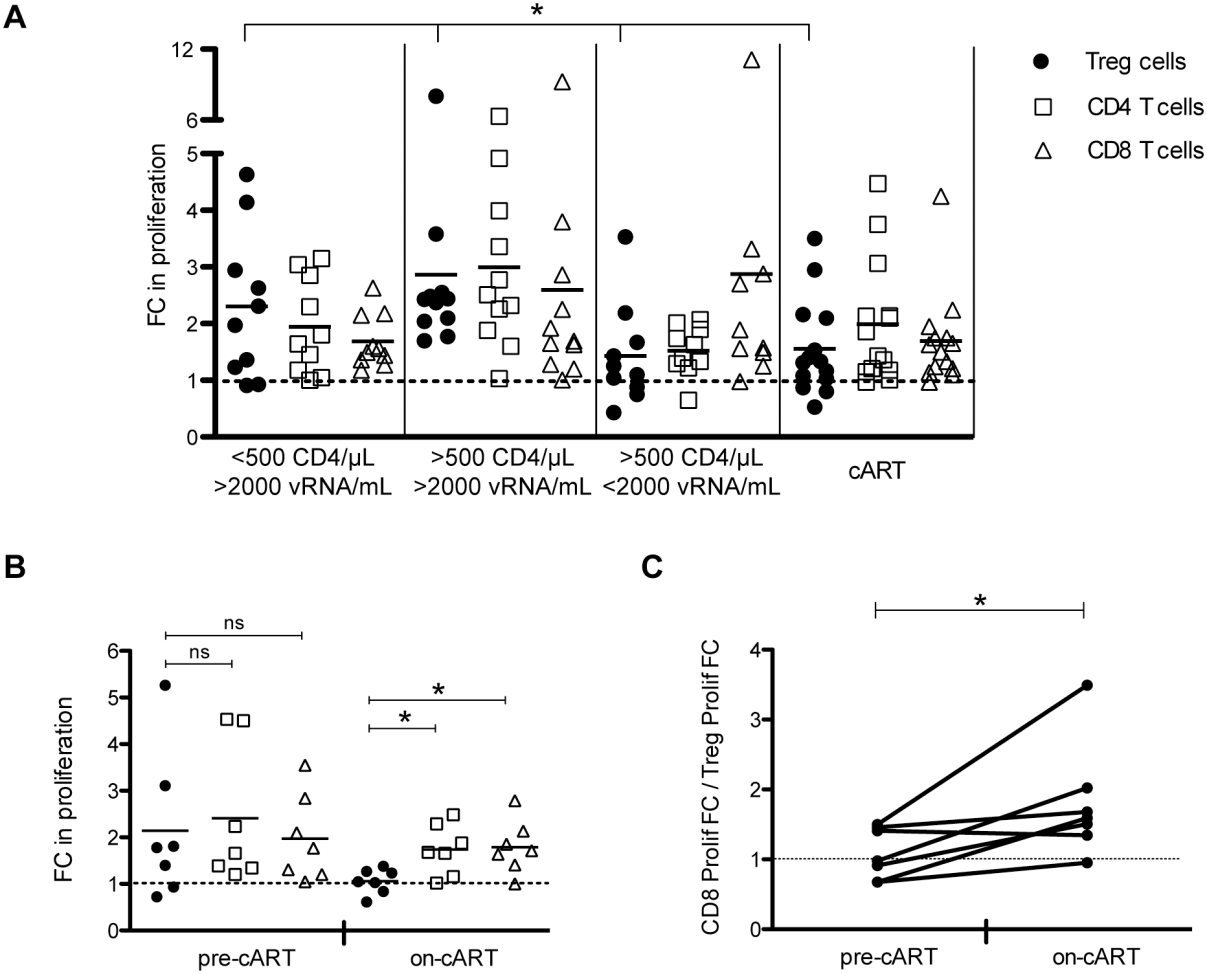
PD-L1 blockade differentially increases Treg and CD8 T cell proliferative capacity depending on host viremia. PBMC from HIV-infected individuals were stimulated with Gag peptides for 6 days in the presence of a PD-L1 blocking antibody or an isotype control antibody. Proliferation was determined by CFSE dilution (A) and alternatively by Ki67 staining (B and C). FC (fold change) in proliferation is calculated as the ratio between PD-L1 blockade conditions and isotype control conditions. Each symbol represents the result for one individual. In panels A and B, the dashed line (FC = 1) indicates no change due to PD-L1 blockade. The means of fold changes in proliferation are shown. (A) Given are the fold changes in the proliferation of Treg (including eTreg and rTreg) (black circles), CD4- (empty squares) and CD8- (empty triangles) T cell populations of different HIV-infected study groups as indicated. Significant differences in fold change of proliferation of Treg, CD4- and CD8- T cells among the 4 HIV study groups were determined using the Kruskal-Wallis test. Significant differences were found in the fold change of proliferation of Treg cells across the 4 HIV- study groups (*p <0.05) but not of CD4- and CD8- T cells. (B) Fold changes in proliferation of Treg, CD4- and CD8- T cell populations upon PD-L1 blockade were measured longitudinally in 7 individuals before and after >2 years of antiretroviral treatment (pre-cART and on-cART, respectively). Significant differences in fold change of proliferation between Treg, CD4- and CD8- T cells were determined by a Wilcoxon matched pairs test (*p <0.05; ns: non significant). (C) Ratio of the FC in proliferation of CD8 T cells and the FC in proliferation of Treg cells upon PD-L1 blockade. The dashed line (FC = 1) indicates the same FC in proliferation for CD8 T and Treg cells upon PD-L1 blockade. Significant differences between the ratios before and after antiretroviral treatment were determined by Wilcoxon matched pairs test (*p <0.05).

To analyse whether the PD-L1 blockade-mediated restoration of the proliferative capacity of Treg, CD4- and CD8- T cells as shown in Fig 5B was dependent on the HIV infection stage of the host, the respective data points were grouped according to viremia, CD4 T cells counts and antiretroviral treatment (patient grouping as of S2 Table). The PD-L1 blockade significantly increased the proliferation of Treg cells from patients with high viremia irrespective of their CD4 T cell counts (Figs 6A and S6B). Treg cells from patients that controlled viremia (either spontaneously or by cART) showed no significant proliferation increase compared with their isotype antibody control stimulation. In contrast, the increase of CD8 T cell proliferation mediated by the PD-L1 blockade was significant with respect to the isotype antibody control for all 4 patient groups (Figs 6A and S6B). Importantly, the PD-L1 blockade affected the Treg cells from the high viremic groups more than the respective CD8 T cells. The inverse was true in the groups with controlled viremia.

To confirm that PD-L1 blockade differentially impacts Treg cells and CD8 T cells depending on the plasma viremia of the host, fold changes in proliferation upon PD-L1 blockade were measured longitudinally in 7 additional individuals before and after antiretroviral treatment (pre-cART and on-cART, respectively). As can be seen in Fig 6B, in samples from pre-cART treatment, PD-L1 blockade increased the proliferative capacity of Treg cells by approximately 2 fold, which is comparable to that of effector CD4- and CD8- T cells. However, in samples from the same patients after a cART period, PD-L1 blockade had no significant effect on Treg cell proliferation. In contrast, the proliferative capacity of effector T cells was increased 2 fold upon PD-L1 blockade comparable to that of pre-cART samples (Fig 6B). As shown in Fig 6C, PD-L1 blockade preferentially increased the proliferative capacity of effector T cells over regulatory T cells in samples from individuals under cART treatment. Thus the net gain of T cell effector function after PD-L1 blockade may critically depend on plasma viremia.

**Fig 7:**
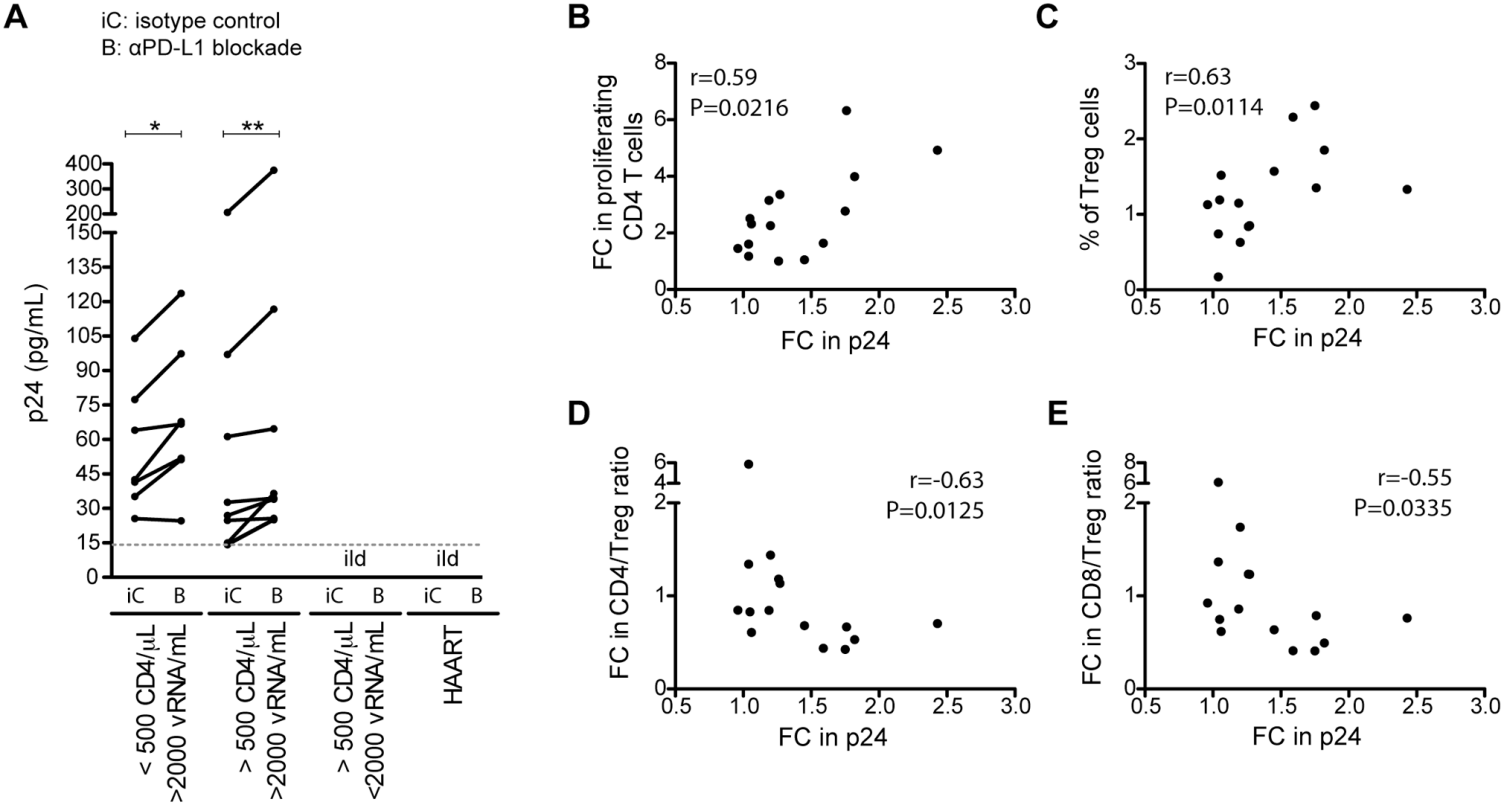
Virus reactivation upon PD-L1 blockade is related to increased percentage of Treg cells. (A) PBMCs were stimulated with Gag peptides in the presence of a PD-L1 blocking antibody or an isotype control antibody. After 4 days in culture, supernatants were harvest to quantify the p24 HIV core antigen by ELISA. The dashed line indicates the ELISA cut-off. Significant differences were determined by a Wilcoxon matched pairs test (*p <0.05; **p <0.01). Ild: inferior to the limit of detection. Panels B to E show correlations between fold change (FC) in p24 and fold change in percentage of proliferating CD4 T cells (B), percentage of Treg cells (including eTreg and rTreg) (C), fold change in the CD4 T cell to Treg cell (CD4/Treg) percentage ratio (D), and fold change in the CD8 T cell to Treg cell (CD8/Treg) percentage ratio (E); respectively. Spearman’s rank correlation coefficients (r) and p values (P) are indicated.

To analyse the consequences of the PD-L1 blockade for *ex vivo* HIV reactivation, supernatants of the above-described PBMC cultures were collected and tested for viral production by HIV p24 antigen determination. HIV production was readily detectable in most PBMC cultures from the viremic patient groups (16 from 19 samples)(Fig 7A). The blockade of PD-L1 consistently increased viral production relative to the isotype antibody control stimulations. This increase of HIV production correlated positively with the increase in CD4 T cell proliferation (Fig 7B) and the fraction of Treg cells in the lymphocyte population (Fig 7C) but not with the increase in Treg cell proliferation (S7 Fig). Furthermore, the increase in HIV production correlated negatively with the fold changes of CD4 T cell/Treg cell as well as CD8 T cell/Treg cell ratios respectively (Fig 7D and 7E). Together this suggests that the inhibitory function of the Treg cells rather than their capacity of being an HIV target cell may play a role in virus expansion under these conditions.

## Discussion

Perhaps the most striking finding of this work is the observation that PD-L1 blockade restores the proliferative capacity of regulatory T cells (Treg cells) from HIV-infected individuals differentially depending on plasma viremia. Treg cells from viremic patients show the largest fold increase in proliferation while the cell´s suppressive capacity is maintained. As Treg cells contribute to maintain exhaustion [48,49], therapeutic interventions aiming to disrupt T cell exhaustion by means of blocking the PD-1 signalling pathway should first reduce the HIV load by antiretroviral drugs. Only this may guarantee the biggest possible net gain of effector T cell function and subsequent better immunological control over HIV.

Relative to healthy controls, the frequency of PD-1-expressing Treg cells was significantly increased in all four groups of HIV-infected individuals and correlated positively with markers of disease progression such as virus load and reduction of CD4 T cells. With this, Treg cells follow the same trend as total CD4- and CD8- T cells (S4 Fig) as previously described [11,13,47]. This is intriguing, as a coordinated up-regulation of a negative signalling receptor on both effector T cells and suppressor T cells seems counterintuitive. However, it is consistent with the model of dynamic co-evolution of memory and regulatory T cells at sites of infection [50,51] and data from subsequent studies of Treg cells in chronic hepatitis C virus infection [34,52]. Accordingly, an expanding virus triggers an effector T cell response with concomitant Treg cell generation. The killing of infected cells by effector T cells then promotes tissue injury. This is dampened via PD-1 signalling on effector cells as well as expanding Treg cells. To limit exaggerated suppression and maintain homeostasis, Treg cell expansion is also controlled by PD-1 on Treg cells. While the data from our cross-sectional study do not enable the analysis of the temporal appearance of the PD-1-expressing T cell subsets in the HIV-infected individuals studied, they are concordant with this model of an antigen-driven coordinated response in order to balance (i) virus reduction by effector T cells and (ii) reduction of immunopathology by Treg cells with (iii) maintenance of the adaptability of T cell responses to subsequent viral bursts.

The direct correlation of PD-1 on Treg cells with patient’s viral load is consistent with the idea that persistent antigen exposure is a main trigger of PD-1 expression. However, exposure of PBMCs to HIV under non-stimulating conditions did not induce PD-1 on effector or on resting Treg cells significantly thus suggesting that additional activation signals are required. Interestingly, this exposure to HIV was sufficient to massively up-regulate PD-L1 on both eTreg and rTreg cells in a virus dose-dependent manner. As PD-L1 can participate in Treg cell induction [26–28] as well as PD-1-mediated suppression [29,30,32], the role of both Treg cell states in virus sensing and subsequent signal conversion merits further investigation.

Upon PD-L1 blockade, the fold change of Treg cell proliferation was the highest in viremic individuals and correlated with viral loads. However the fold change of Treg cell proliferation was not significantly correlated with PD-1 expression itself although PD-1 expression was correlated to viral loads. Thus there might be additional factors that have participated in the observed gain in proliferation. One good candidate for this is IL-2 that is produced upon T cell stimulation but may have been limiting with increasing PD-1 expression and negative signalling on CD4 T cells, the main IL-2 producers. Consistent with this are previous observations that (i) the PD-1/PD-L1 pathway negatively regulates Treg cell proliferation by inhibiting the IL-2 signalling cascade [34] and (ii) exogenous IL-2 can overcome PD-1/PD-L1-mediated inhibition of proliferation [53].

The PD-L1 blockade of PBMC from viremic HIV-infected individuals under stimulating conditions commonly led to increased reactivation of HIV *ex vivo*. The observed correlations between the fold change in virus p24 with the fold change in proliferating CD4 T cells, the percentage of Treg cells as well as the negative correlations with the fold changes of CD4 and CD8 to Treg cell ratios respectively, may indicate that an increase of Treg cells in relation to CD8 T cells promotes virus expansion. These observations are compatible with the characteristics of HIV biology and stress the importance of applying antiviral treatment in addition to the PD-L1 blockade therapy.

In summary, this *ex-vivo* study of Treg cell behaviour from different HIV-infected patient groups demonstrates (i) an up-regulation of PD-1 and PD-L1 that correlates with markers of disease progression and (ii) a differential and plasma viremia-dependent gain of Treg cell proliferation and overall suppressive function upon PD-L1 blockade. This has direct consequences for patient selection to enter clinical trials targeting the PD-1/PD-L1 signalling pathway and treatment modalities. While the translation of our results to HIV infection *in vivo* is complex, and many aspects about the weight of the possible PD-1/PD-L1 roles for HIV expansion at different anatomical sites are not completely defined, the upcoming clinical trials will definitely increase our knowledge on Treg cell biology and provide a clear picture on the pros and cons of immune checkpoint modification in HIV infection.

## Materials and Methods

### Human subjects and PBMC isolation

Blood was obtained from healthy, HIV-uninfected volunteers and HIV-infected individuals at the Hospital Clinic and the Hospital del Mar, both in Barcelona, Spain. HIV-infected individuals were categorized into 4 groups: (1) fewer than 500 CD4/μL and more than 2000 RNA copies/mL blood; (2) more than 500 CD4/μL and more than 2000 RNA copies/mL blood; (3) more than 500 CD4/μL and fewer than 2000 RNA copies/mL blood; (4) HIV-infected patients under successful antiretroviral treatment (cART) for at least 2 years with more than 500 CD4/μL blood and viral loads below the limit of detection (40 RNA copies/mL blood). HIV-infected individuals from the cross-sectional study with the exception of the cART group were naïve to antiretroviral therapy at the time of testing and were not in the primary infection phase (S1 and S2 Tables). In addition, we studied two subgroups of patients longitudinally. A group of 5 individuals were followed before starting cART, during cART, upon interruption of cART, and after restarting cART (S3 Table, group A). A second group of 7 individuals were followed before cART and after 2 years receiving cART (S3 Table, group B). Peripheral blood mononuclear cells (PBMC) were isolated by Ficoll density centrifugation (Invitrogen) and frozen for subsequent analyses.

### Ethics statement

Ethical committee approval and written informed consent from all subjects, in accordance with the Declaration of Helsinki, were obtained prior to study initiation. The study was approved by the institutions’ ethical committees: CEIC- Parc de Salut Mar, Barcelona, Spain (Protocol approval number: 2013/5422/I) and Comitè étic d'investigació clínica, Hospital Clinic, Barcelona, Spain (Protocol approval numbers: 2013/8671 and 2008/4575 amendment version 1.0 from 13/03/2013).

### Flow cytometry

PBMC were stained with the Live/Dead fixable violet dye (Invitrogen) and the following fluorochrome-labelled monoclonal antibodies: CD3-BV605 (clone SK7), CD3-PerCPCy5.5 (clone SK7), CD4-PECy7 (clone SK4), CD4-APCCy7 (clone SK3), CD8-PE (clone RPA-T8), CD8-APCCy7 (clone SK1), CD25-APCH7 (clone M-A251), CD127-PE (clone HIL-7R-M21), CD45RA-FITC (clone HI100), CD45RA-PerCPCy5.5 (clone HI100), CD45RA-eFluor605 (eBioscience, clone HI100), PD-1-PerCPCy5.5 (clone EH12.1), PD-L1-PE (clone MIH1), CD39-PECy7 (eBioscience, clone eBiosA1) and CTLA4-PE (clone BNI3), FOXP3-Alexa647 (clone 259D/C7), Helios-PerCPCy5.5 (clone 22F6) and Ki67-PE (clone B56). For intracellular detection of FOXP3, Helios and Ki67, cells were fixed and permeabilized using the FOXP3 staining kit (eBioscience) according to manufacturer’s instructions. All antibodies were from BD Biosciences unless otherwise stated.

Flow cytometry data were collected on a LSR Fortessa (BD biosciences) and analysed with Flow Jo software (Tree Star). Panels containing the corresponding isotype controls were collected to set PD-1, PD-L1, CTLA4 and Ki-67 gates. Treg cells were identified as a joint population of effector Treg cells (CD4+CD45RA-FOXP3^hi^) (eTreg) and resting Treg cells (CD4+CD45RA+FOXP3lo) (rTreg), in which the cut offs for FOXP3 were set manually in relation to CD45RA expression as previously described [40] (Figs 1 and S1A).

To verify that the CD4+CD45RA+FOXP3lo & CD4+CD45RA-FOXP3hi cell populations define Treg cells after a 6-day culture, both rTreg cells (CD4+ CD127lo CD25+ CD45RA+) and conventional CD4 T cells (CD4+ CD127+ CD25-) were isolated from HIV-infected individuals by flow cytometry, labelled with CFSE and cultured in the presence of autologous, unlabelled PBMC and Gag peptides as described below. For this, PBMC samples were enriched for CD4 T cells using magnetic beads (Miltenyi Biotec) and sorted by an ARIA SORP (BD biosciences) (S1B Fig).

### PBMC exposure to HIV

HIV-1Bal was obtained from the Centre for AIDS Reagents NIBSC (repository reference: ARP118) and propagated in PHA-stimulated PBMC in RPMI-1640 media (Gibco) supplemented with 20% FBS (Sigma), 1% penicillin/ streptomycin (Gibco) and 10U/mL rhIL-2 (R&D Systems) for 7 days. Supernatants were collected, titrated on TZM-bl cells and frozen at -80°C until use. Supernatants from non-infected PBMC cultured under identical conditions were collected as mock control.

PBMC from healthy controls were cultured in the presence of HIV-1Bal (or mock) at a multiplicity of infection (MOI) of 0.3 and 0.03. To discard that PD-L1 upregulation requires HIV infection, PBMC were infected at MOI 0.3 in the presence or absence of 5 μM T20 HIV entry inhibitor. In parallel, PBMC were also cultured with HIV-1 Bal gp120 (NIH reagent program catalogue number 4961) at 0.01ng/mL and 1ng/mL. After 4 hours, HIV-exposed cells were washed twice while gp120-exposed cells were left as such. 0.5·10^6^ PBMC/well were cultured in 48-well plates in RPMI-1640 media (Gibco) supplemented with 10% FBS (Sigma), 1% penicillin/ streptomycin (Gibco). After 3 days, cells were harvested and stained to analyse PD-1 and PD-L1 expression on Treg cells by flow cytometry.

The efficacy of HIV-1Bal inhibition by T20 treatment was controlled by stimulating the virus-exposed cells from above with 5μg/ml PHA at day 3 and culturing them for further 7 days. The presence or absence of virus production was determined by a p24 HIV core antigen ELISA kit (Innogenetics). T20 treatment completely blocked HIV-1Bal infection under these conditions.

### Cell culture and proliferation assay

2·10^6^ PBMC/well were cultured in 24-well plates in RPMI-1640 media (Gibco) supplemented with 10% FBS (Sigma), 1% penicillin/ streptomycin (Gibco) and 1μg/mL anti-CD28 and anti-CD49d antibodies. Cells were either left unstimulated or incubated with 1μg/mL Gag pool of overlapping peptides (Gag peptides; NIH reagent program catalogue number 8117 and 8118, in part kindly provided by Anja Germann and Hagen von Briesen, Fraunhofer IBMT, Germany) plus 5μg/mL anti-PD-L1 blocking or isotype control antibodies (eBioscience).

For proliferation assays, PBMC were stained with Carboxyfluorescein succinimidyl ester (CFSE) (Invitrogen) as described in (Quah et al., Nature Protocols, 2007). After 6-day-culture cells were harvested and stained to analyse proliferation of Treg, CD4- and CD8- T cell subsets. Alternatively, to analyse proliferation after a 6-day culture in longitudinal samples for which cell numbers were limited, non-CFSE stained PBMC were cultured as previously described, and stained with Ki67 or isotype control antibodies. Fold change in proliferation (FC proliferation) was calculated as a ratio of proliferation under PD-L1 blockade condition divided by proliferation under control condition.

### *In vitro* suppression assay for functional assessment of regulatory T cells

PBMC were stimulated with Gag peptides in the presence of anti-PD-L1 blocking antibody or isotype control antibody as described above. After 6-day culture, Treg cells where isolated by magnetic beads using the CD4+CD25+CD127dim/- regulatory T cell isolation kit II (Mitenyi Biotec; Treg cell purity shown in S5 Fig). Purified Treg cells were co-cultured with 50.000 CFSE-labelled PBMC at different ratios and stimulated with 0.5μg/mL anti-CD3 and 1μg/mL anti-CD28 antibodies. Cells were cultured in 96-U bottom well plates (Greiner bio-one) in RPMI-1640 media (Gibco) supplemented with 10% FBS (Sigma), 1% penicillin/ streptomycin (Gibco), 50U/mL rhIL-2 (R&D Systems) and 1mM sodium pyruvate (Sigma). After a 3-day-culture, cells were harvested and stained to analyse proliferation of CD8 T cells. The Treg cell suppressive capacity was determined by the percentage of inhibition of CD8 proliferation, calculated as: [(CD8 proliferation − CD8 proliferation in presence of Treg cells) / CD8 proliferation] x 100.

### ELISA

Culture supernatants after a 4-day culture in the presence of Gag peptides and anti-PD-L1 blocking or isotype control antibodies (as described in previous section “Cell culture and proliferation assay”) were collected for p24 HIV core antigen quantification by an ELISA kit (Innogenetics).

### Statistical analysis

Comparisons between two groups were performed using Mann-Whitney *U* test, between more than two groups using Kruskal-Wallis test and within the same patient using Wilcoxon matched pairs test. Bonferroni correction was applied to adjust significance for multiple comparisons. Correlation coefficients (r) were calculated using the Spearman rank correlation test. Categorical variables between study groups were compared using Chi-squared and Fisher’s exact test.

Statistical analyses were performed using GraphPad Prism 5.0 (San Diego, CA, USA) and SPSS 15.0 statistical software (Chicago, IL, USA). *p*-values (P) below 0.05 were considered significant and were indicated by asterisks: * p<0.05; ** p<0.01; *** p<0.001. Non-significant differences were indicated as “ns”.

### Accession numbers

Accession numbers in Uniprot database for proteins mentioned in the text are: PD-1 (Q15116), PD-L1 (Q9NZQ7), CD3 (P07766), CD4 (P01730), CD8 (P01732), CD25 (P01589), CD45RA (P08575), FOXP3 (Q9BZS1), CD127 (P16871), CD39 (P49961), CTLA4 (P16410), Helios (Q9UKS7), Ki67 (P46013), Gag (Q73367), IL-2 (P60568), CD28 (P10747) and CD49d (P13612).

## Supporting Information

S1 Table Comparison of demographic characteristics of HIV-infected individuals and healthy controls used in Fig 1. (PDF)

S2 Table Characteristics of HIV-infected individuals. (PDF)

S3 Table Viral loads and CD4 T cell counts from HIV-infected individuals studied longitudinally. (PDF)

S1 Fig Gating strategy for regulatory T cells. (A) Representative gating of resting Treg and effector Treg cells. (B) Verification of Treg cell gating strategy after 6-day stimulation with Gag peptides. rTreg cells and conventional CD4 T cells were sorted via CD4+CD127-CD25+CD45RA+ and CD4+CD127+CD25- markers respectively. Then cells were labelled with CFSE and cultured in the presence of non-labelled, autologous PBMC in a ratio of 1:30, and stimulated with Gag peptides. CFSE-labelled cells were analysed after 6-day stimulation. Dot plots showing the gating of rTreg and eTreg cells (left) and proliferation of CFSE-labelled cells (right) from one representative example of 2 donors.
(TIF)

S2 Fig Absolute numbers and percentages of regulatory T cells. Given are total as well as effector and resting Treg cell counts/μL blood (up) and percentages from CD4 T cells (down) from different HIV-infected study groups as indicated. The absolute numbers were calculated from the percentage of regulatory T cells among the CD4 T cells and the CD4 T cell counts for each HIV-infected individual. The mean ± SEM (standard error of the mean) is shown.
(TIF)

S3 Fig HIV exposure induces PD-L1 on Treg cells. Shown are the percentages of PD-L1 expression on Treg cells for different conditions and individuals. Each graph represents one individual. (A) PBMC from 3 healthy controls exposed to HIV-1 Bal at 0.03 and 0.3 (black bars) multiplicity of infection, compared with mock controls (white bars). (B) PBMC from 3 healthy controls exposed to HIV-1 Bal at 0.3 multiplicity of infection in the absence (black bars) or in the presence (stripped bars) of the HIV entry inhibitor T20. (C) PBMC from 3 healthy controls exposed to HIV gp120 at 2 different concentrations.
(TIF)

S4 Fig PD-1 expression on CD4- and CD8- T cells. (A) Percentages of PD-1-expressing CD4- and CD8- T cells from HIV-infected individuals (black circles) and healthy controls (empty triangles) are shown. The mean ± SEM (standard error of the mean) is shown. Significant differences were determined by a Mann-Whitney U test, corrected for multiple comparisons using the Bonferroni method, and indicated by asterisks (*p <0.05; **p <0.01). (B) Correlations of PD-1 expression on CD4- and CD8- T cells with viral loads and CD4 T cell counts are shown, respectively. Each dot represents the result from one individual. Spearman’s rank correlation coefficients (r) and p values (P) are given for each correlation.
(TIF)

S5 Fig Purity of isolated Treg cells. Purity of isolated Treg cells used for the suppressive assays as shown in Fig 5F. There were no significant differences in the purity of the Treg cells expanded under control conditions or PD-L1 blockade conditions. (A) A representative flow cytometry dot plot showing the percentage of rTreg, eTreg and CD45RA-FOXP3lo T cells before (left) and after (right) isolating Treg cells with a commercial kit for CD4+CD25hiCD127lo cell isolation. Purity of Treg cells after isolation from the control culture (upper right) and the PD-L1 blockade culture (lower right) is shown. (B) Raw data of contaminating CD45RA-FOXP3lo T cells for the Treg cell isolations used to determine the Treg cell suppressive capacity as shown in Fig 5F.
(TIF)

S6 Fig Impact of PD-L1 blockade for Treg, CD4- and CD8- T cells. This figure is another representation of the data of Figs 5E and 6A (differences rather than ratios are shown). PBMC from HIV-infected individuals were stimulated with Gag peptides for 6 days in the presence of a PD-L1 blocking antibody or an isotype control antibody. Significant differences between PD-L1 blockade and isotype control conditions were determined by a Wilcoxon matched pairs test (*p <0.05; **p <0.01; ***p <0.001; ns: non significant). (A) Percentages of CD39, CTLA4 and Helios on Treg cells are shown. (B) Percentages of proliferating Treg cells, CD4- and CD8- T cells determined by CFSE dilution are shown.
(TIF)

S7 Fig Lack of correlation between FC in p24 and FC in percentage of proliferating Treg cells. PBMCs were stimulated with Gag peptides in the presence of a PD-L1 blocking antibody or an isotype control antibody. After 4 days in culture, supernatants were harvest to quantify the p24 HIV core antigen by ELISA. Correlation between fold change in p24 and fold change in percentage of proliferating Treg cells cells is shown. Spearman’s rank correlation coefficient (r) and p value (P) are indicated.
(TIF)
