# Supplementary material for: PD-L1 Blockade Differentially Impacts Regulatory T Cells from HIV-Infected Individuals Depending on Plasma Viremia
Source: PLoS Pathog. 2015 Dec 3;11(12):e1005270. doi: 10.1371/journal.ppat.1005270 (PMC4669187; doi:10.1371/journal.ppat.1005270)
Supplement: S1 Table — (PDF) [file ppat.1005270.s001.pdf]

S1 Table. Comparison of demographic characteristics of HIV-infected individuals and healthy controls used in Fig 1

	**for PD-1 staining**			**for PD-L1 staining**		
	**HIV patients** n= 46	**Healthy Controls** n= 9	**p-value**[a]	**HIV patients** n= 23	**Healthy Controls** n= 7	**p-value**[a]
**age** (years) mean ± SD	39.43± 8.65	36.11± 12.81	0,3389	37.22± 8.92	39.71± 11.64	0,539
**Sex**, male: female	33:13	3:6	NA	21:2	4:3	NA

[a] p-values were calculated by Mann-Whitney test

NA not applicable
