# Supplementary material for: PD-L1 Blockade Differentially Impacts Regulatory T Cells from HIV-Infected Individuals Depending on Plasma Viremia
Source: PLoS Pathog. 2015 Dec 3;11(12):e1005270. doi: 10.1371/journal.ppat.1005270 (PMC4669187; doi:10.1371/journal.ppat.1005270)
Supplement: S2 Table — (PDF) [file ppat.1005270.s002.pdf]

S2 Table. Characteristics of HIV-infected individuals

	**< 500 CD4/µL > 2000 vRNA/mL** n=11	**> 500 CD4/µL > 2000 vRNA/mL** n=11	**> 500 CD4/µL < 2000 vRNA/mL** n=10	**HAART** n=14	**p-value**[a]
**age** (years) mean ± SD	32.55 ± 5.3	42.09 ± 8.5	39.3 ± 9.6	42.86 ± 7.6	0,0367
**Sex**, male: female	10:1	7:4	8:2	8:6	NA
**CD4 count** (cells/µL) Median, IQR (25-75%)	267 (150-365)	624 (543-696)^	704 (594-787)^	780 (606-869)^	<0.0001
**viral RNA** (LOG10 copies/mL) Median, IQR (25-75%)	4.69 (4.32-4.9) +	4.48 (3.6-5.1) +	2.3 (1.89-2.48) ++	1.57 (1.57-1.57) ++	<0.0001
**Hepatitis C**, positive: negative	3:8	0:11	0:10	4:10	NA
**Hepatitis B**, positive: negative	01:10	00:11	00:10	01:13	NA

[a] p-values were calculated by Kruskal-Wallis to compare the 4 HIV study groups

^ non significant differences in CD4 count between the indicated groups (Kruskal Wallis test , p-value:0.3371)

+ non significant differences in viral RNA between the indicated groups (Mann Whitney test, p-value: 0.5326)

++ significant differences in viral RNA between the indicated groups (Mann Whitney test, p-value: <0.0001)

NA not applicable

vRNA: viral RNA
