# Supplementary material for: PD-L1 Blockade Differentially Impacts Regulatory T Cells from HIV-Infected Individuals Depending on Plasma Viremia
Source: PLoS Pathog. 2015 Dec 3;11(12):e1005270. doi: 10.1371/journal.ppat.1005270 (PMC4669187; doi:10.1371/journal.ppat.1005270)
Supplement: S3 Table — (PDF) [file ppat.1005270.s003.pdf]

S3 Table. Viral loads and CD4 T cell counts from HIV-infected individuals studied longitudinally

Group A		**CD4 count** (cells/µL)	**viral RNA** (copies/mL)
Individual 1	pre-cART	1169	1850
	on-cART	1704	199
	off-cART	566	22984
	on-cART	1119	37
Individual 2	pre-cART	968	110000
	on-cART	941	19
	off-cART	962	4817
	on-cART	966	37
Individual 3	pre-cART	809	4347
	on-cART	1100	199
	off-cART	595	89300
	on-cART	337	37
Individual 4	pre-cART	462	976
	on-cART	1331	199
	off-cART	885	100000
	on-cART	1744	37
Individual 5	pre-cART	440	7160
	on-cART	577	199
	off-cART	427	25200
	on-cART	956	37

Group B		**CD4 count** (cells/µL)	**viral RNA** (copies/mL)
Individual 1	pre-cART	550	32061
	on-cART	524	37
Individual 2	pre-cART	605	4966
	on-cART	1006	37
Individual 3	pre-cART	552	33900
	on-cART	775	37
Individual 4	pre-cART	488	265000
	on-cART	434	37
Individual 5	pre-cART	574	309
	on-cART	1872	199
Individual 6	pre-cART	968	110000
	on-cART	941	19
Individual 7	pre-cART	1169	1850
	on-cART	1704	199
