# Supplementary figures and images for: PD-L1 Blockade Differentially Impacts Regulatory T Cells from HIV-Infected Individuals Depending on Plasma Viremia
Source: PLoS Pathog. 2015 Dec 3;11(12):e1005270. doi: 10.1371/journal.ppat.1005270 (PMC4669187; doi:10.1371/journal.ppat.1005270)

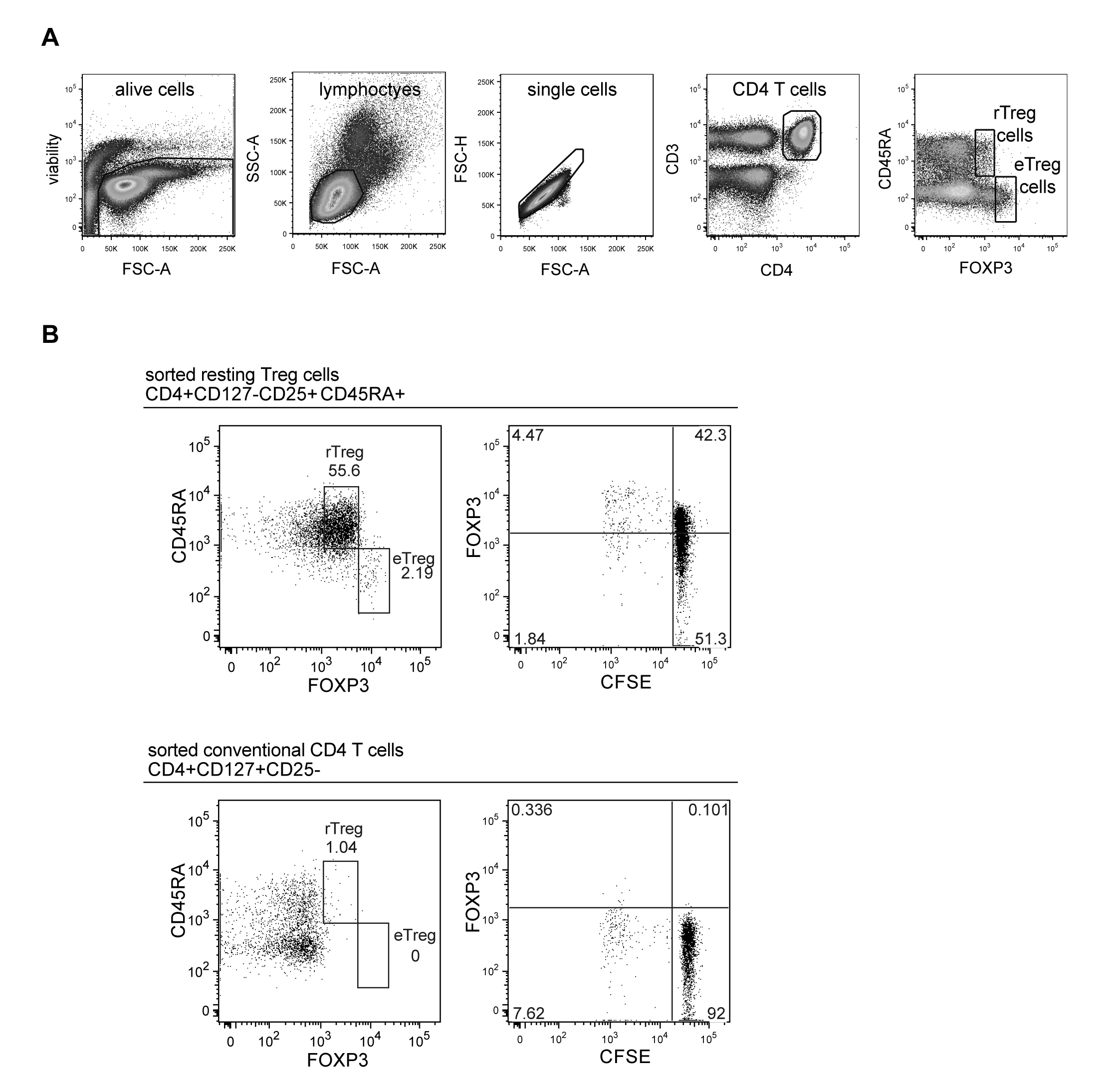

Supplement: S1 Fig — (A) Representative gating of resting Treg and effector Treg cells. (B) Verification of Treg cell gating strategy after 6-day stimulation with Gag peptides. rTreg cells and conventional CD4 T cells were sorted via CD4+CD127-CD25+CD45RA+ and CD4+CD127+CD25- markers respectively. Then cells were labelled with CFSE and cultured in the presence of non-labelled, autologous PBMC in a ratio of 1:30, and stimulated with Gag peptides. CFSE-labelled cells were analysed after 6-day stimulation. Dot plots showing the gating of rTreg and eTreg cells (left) and proliferation of CFSE-labelled cells (right) from one representative example of 2 donors. (TIF) [file ppat.1005270.s004.tif]

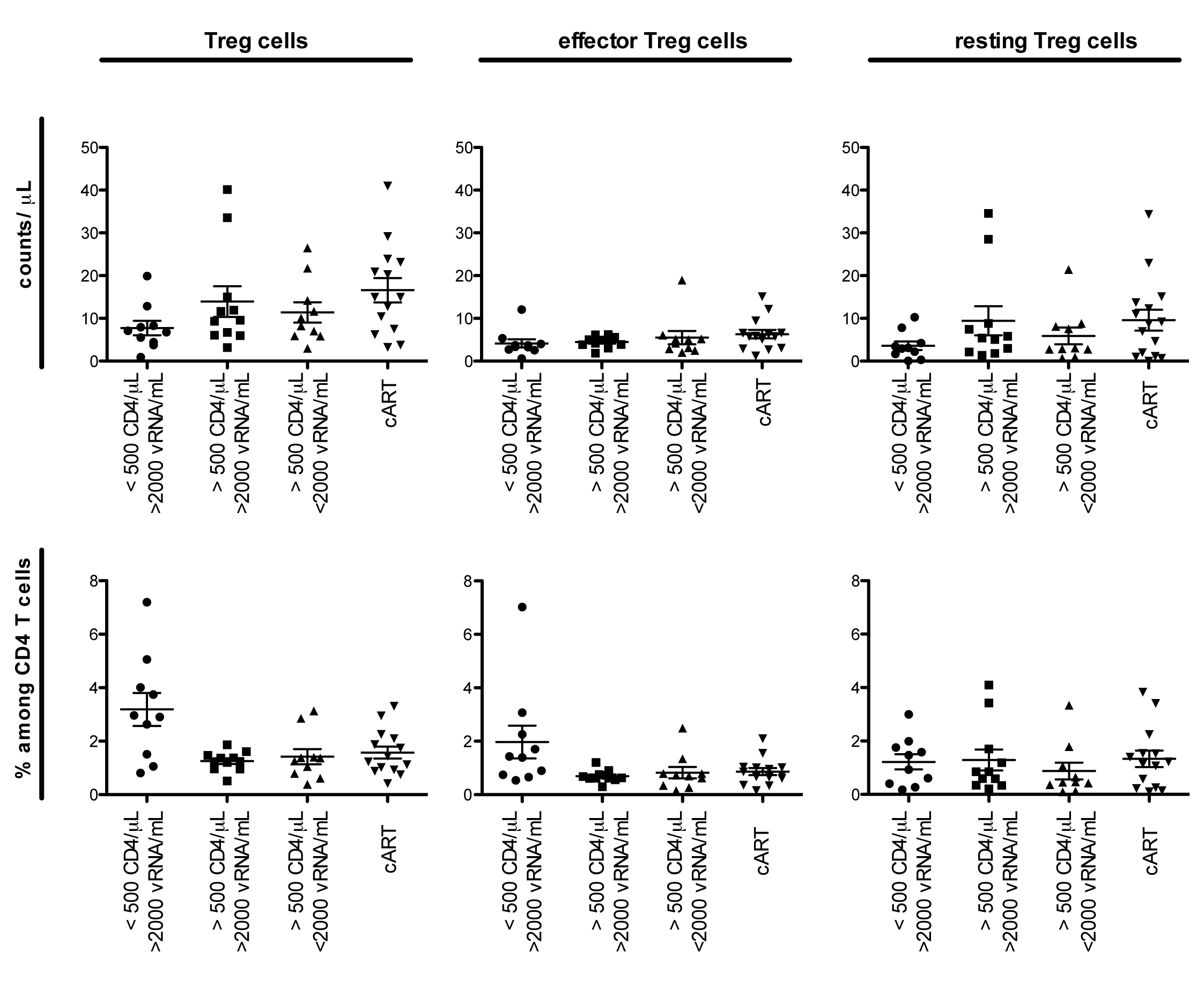

Supplement: S2 Fig — Given are total as well as effector and resting Treg cell counts/μL blood (up) and percentages from CD4 T cells (down) from different HIV-infected study groups as indicated. The absolute numbers were calculated from the percentage of regulatory T cells among the CD4 T cells and the CD4 T cell counts for each HIV-infected individual. The mean ± SEM (standard error of the mean) is shown. (TIF) [file ppat.1005270.s005.tif]

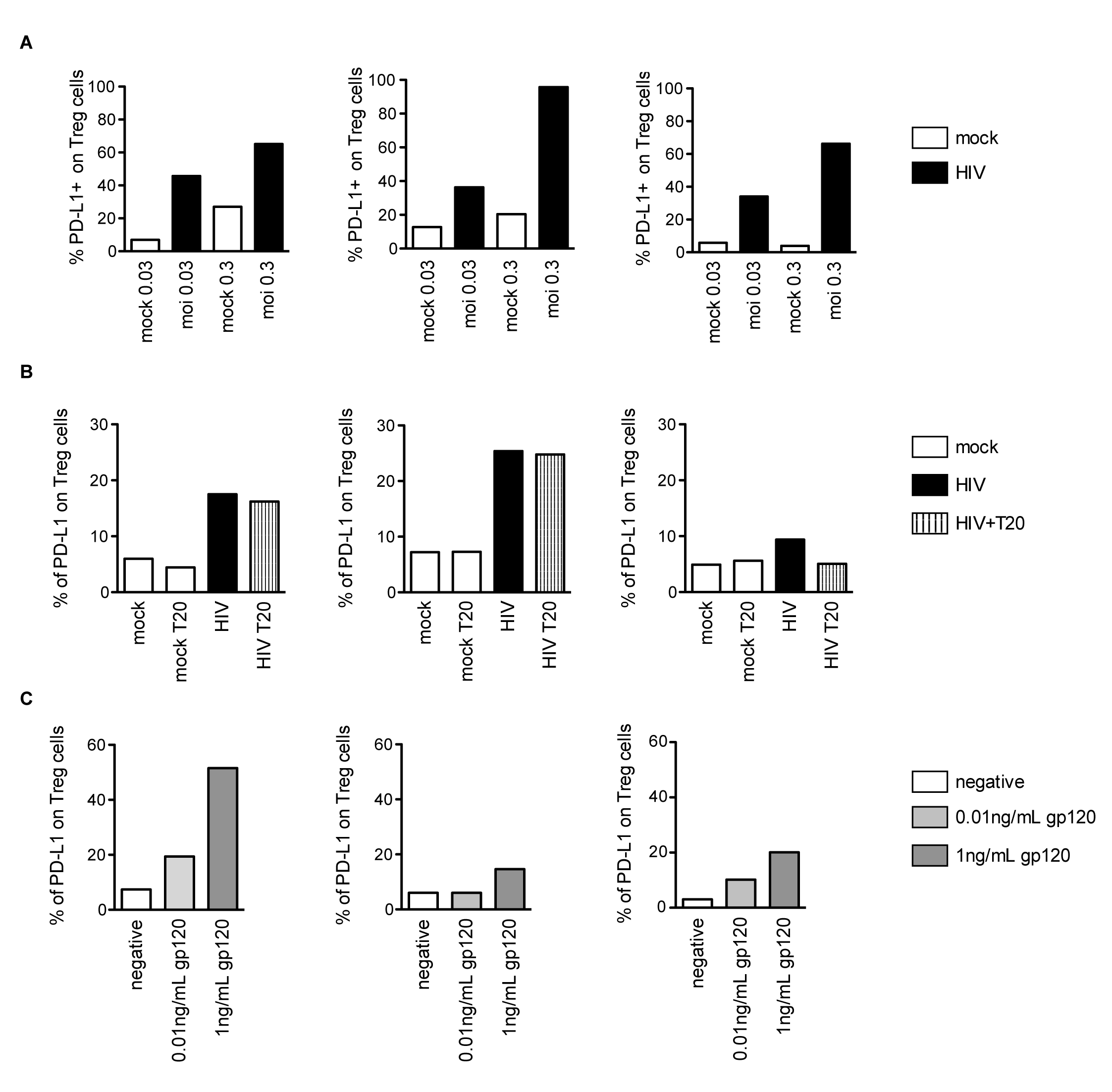

Supplement: S3 Fig — Shown are the percentages of PD-L1 expression on Treg cells for different conditions and individuals. Each graph represents one individual. (A) PBMC from 3 healthy controls exposed to HIV-1 Bal at 0.03 and 0.3 (black bars) multiplicity of infection, compared with mock controls (white bars). (B) PBMC from 3 healthy controls exposed to HIV-1 Bal at 0.3 multiplicity of infection in the absence (black bars) or in the presence (stripped bars) of the HIV entry inhibitor T20. (C) PBMC from 3 healthy controls exposed to HIV gp120 at 2 different concentrations. (TIF) [file ppat.1005270.s006.tif]

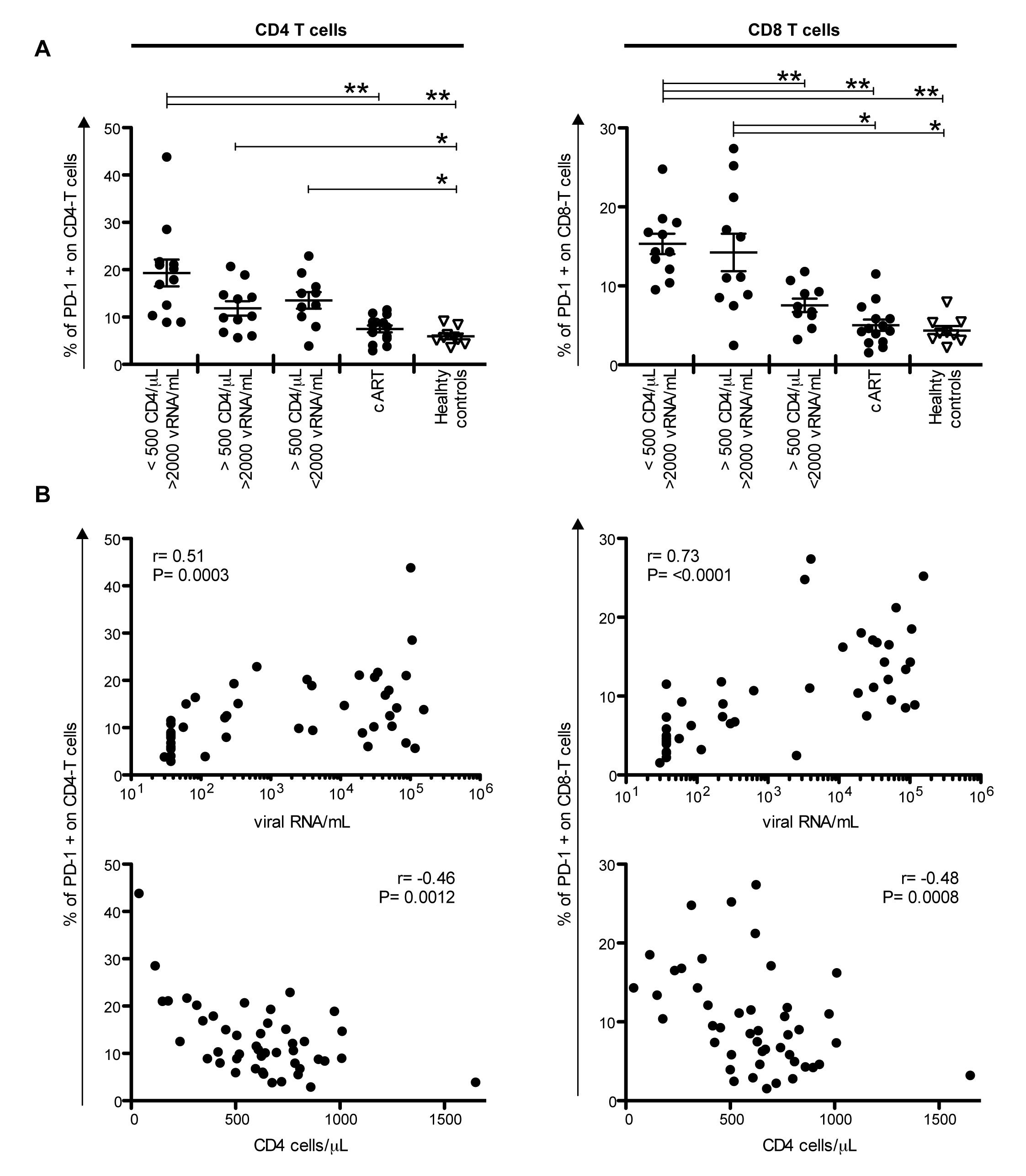

Supplement: S4 Fig — (A) Percentages of PD-1-expressing CD4- and CD8- T cells from HIV-infected individuals (black circles) and healthy controls (empty triangles) are shown. The mean ± SEM (standard error of the mean) is shown. Significant differences were determined by a Mann-Whitney U test, corrected for multiple comparisons using the Bonferroni method, and indicated by asterisks (*p <0.05; **p <0.01). (B) Correlations of PD-1 expression on CD4- and CD8- T cells with viral loads and CD4 T cell counts are shown, respectively. Each dot represents the result from one individual. Spearman’s rank correlation coefficients (r) and p values (P) are given for each correlation. (TIF) [file ppat.1005270.s007.tif]

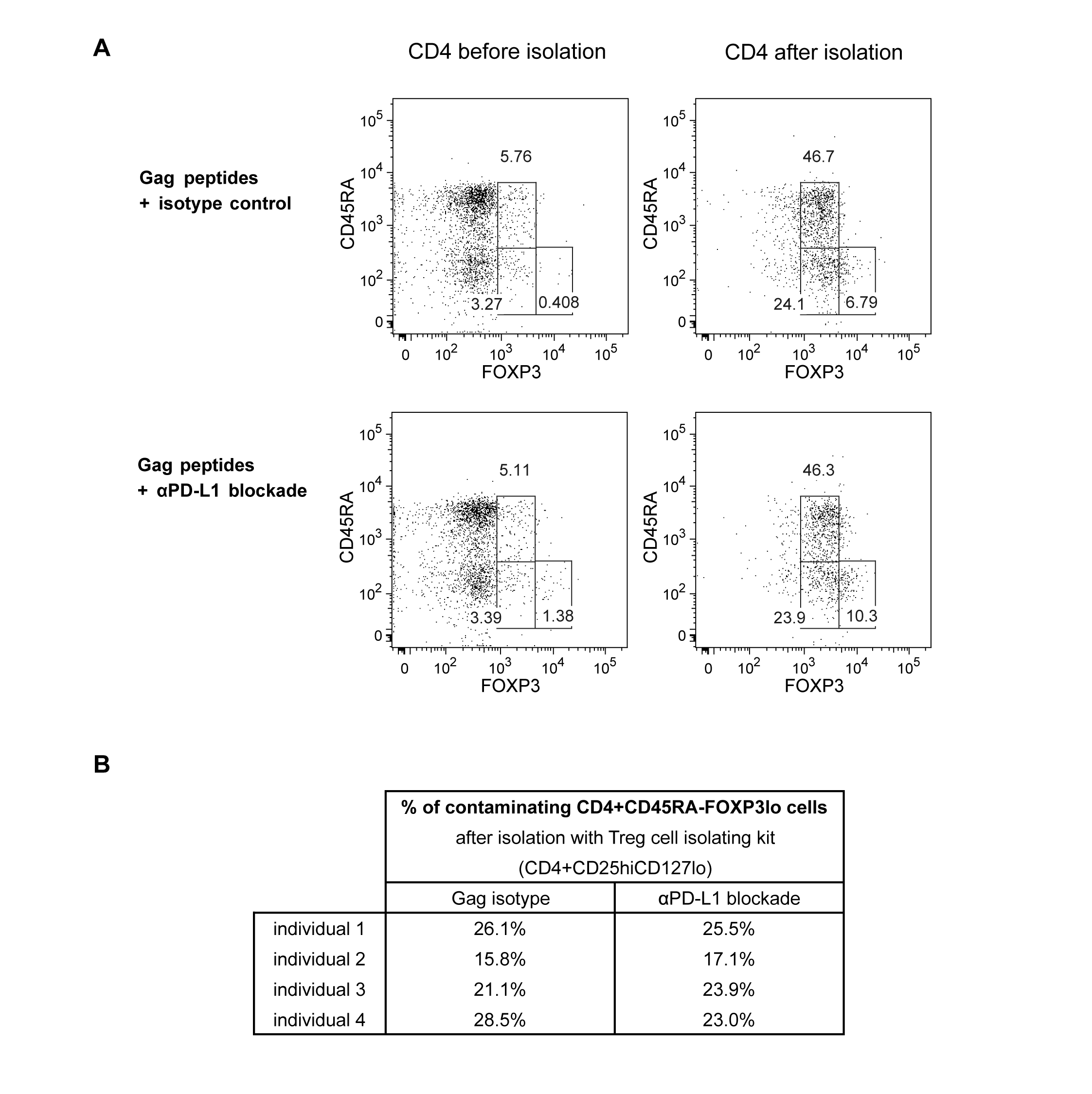

Supplement: S5 Fig — Purity of isolated Treg cells used for the suppressive assays as shown in Fig 5F. There were no significant differences in the purity of the Treg cells expanded under control conditions or PD-L1 blockade conditions. (A) A representative flow cytometry dot plot showing the percentage of rTreg, eTreg and CD45RA-FOXP3lo T cells before (left) and after (right) isolating Treg cells with a commercial kit for CD4+CD25hiCD127lo cell isolation. Purity of Treg cells after isolation from the control culture (upper right) and the PD-L1 blockade culture (lower right) is shown. (B) Raw data of contaminating CD45RA-FOXP3lo T cells for the Treg cell isolations used to determine the Treg cell suppressive capacity as shown in Fig 5F. (TIF) [file ppat.1005270.s008.tif]

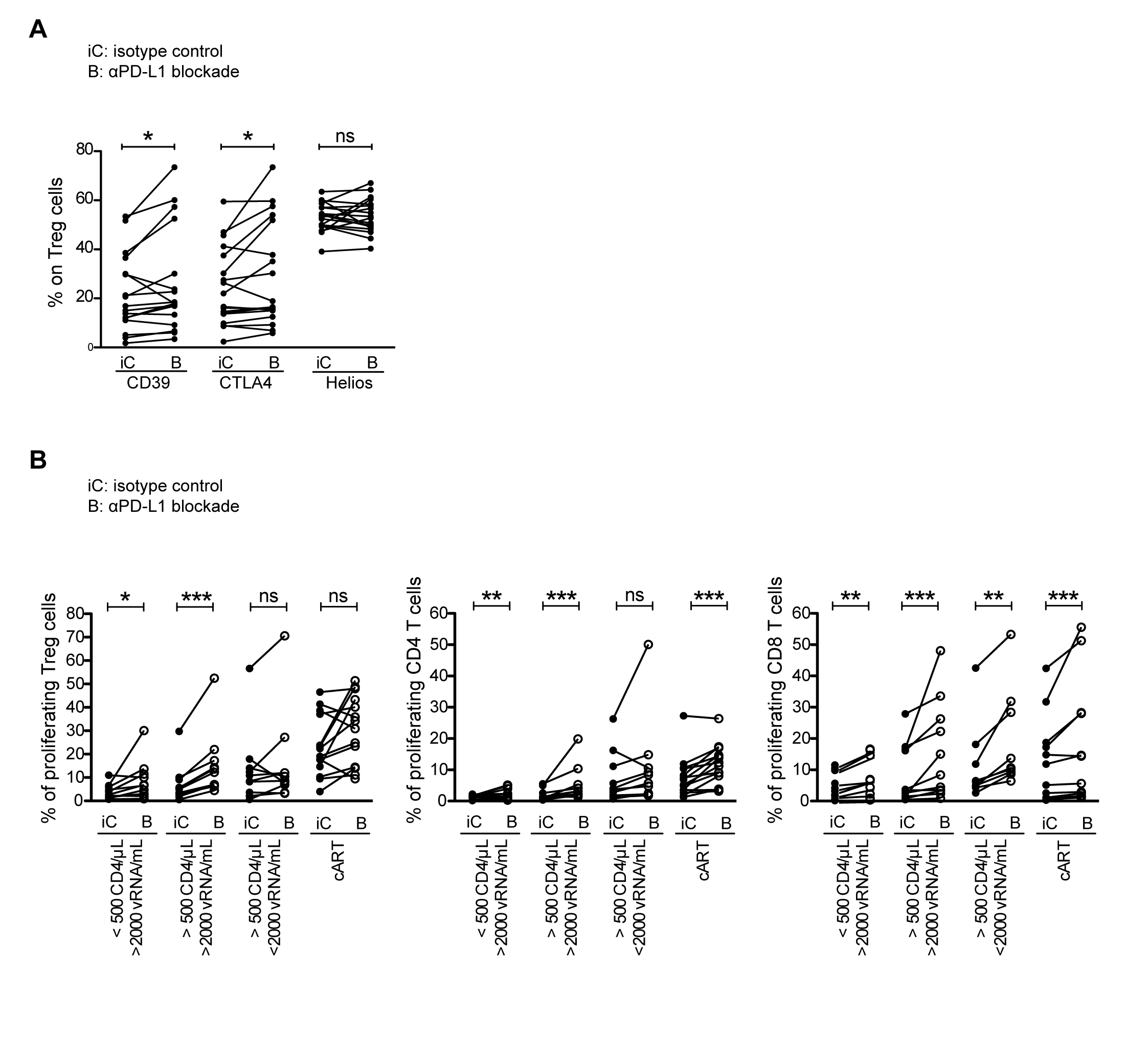

Supplement: S6 Fig — This figure is another representation of the data of Figs 5E and 6A (differences rather than ratios are shown). PBMC from HIV-infected individuals were stimulated with Gag peptides for 6 days in the presence of a PD-L1 blocking antibody or an isotype control antibody. Significant differences between PD-L1 blockade and isotype control conditions were determined by a Wilcoxon matched pairs test (*p <0.05; **p <0.01; ***p <0.001; ns: non significant). (A) Percentages of CD39, CTLA4 and Helios on Treg cells are shown. (B) Percentages of proliferating Treg cells, CD4- and CD8- T cells determined by CFSE dilution are shown. (TIF) [file ppat.1005270.s009.tif]

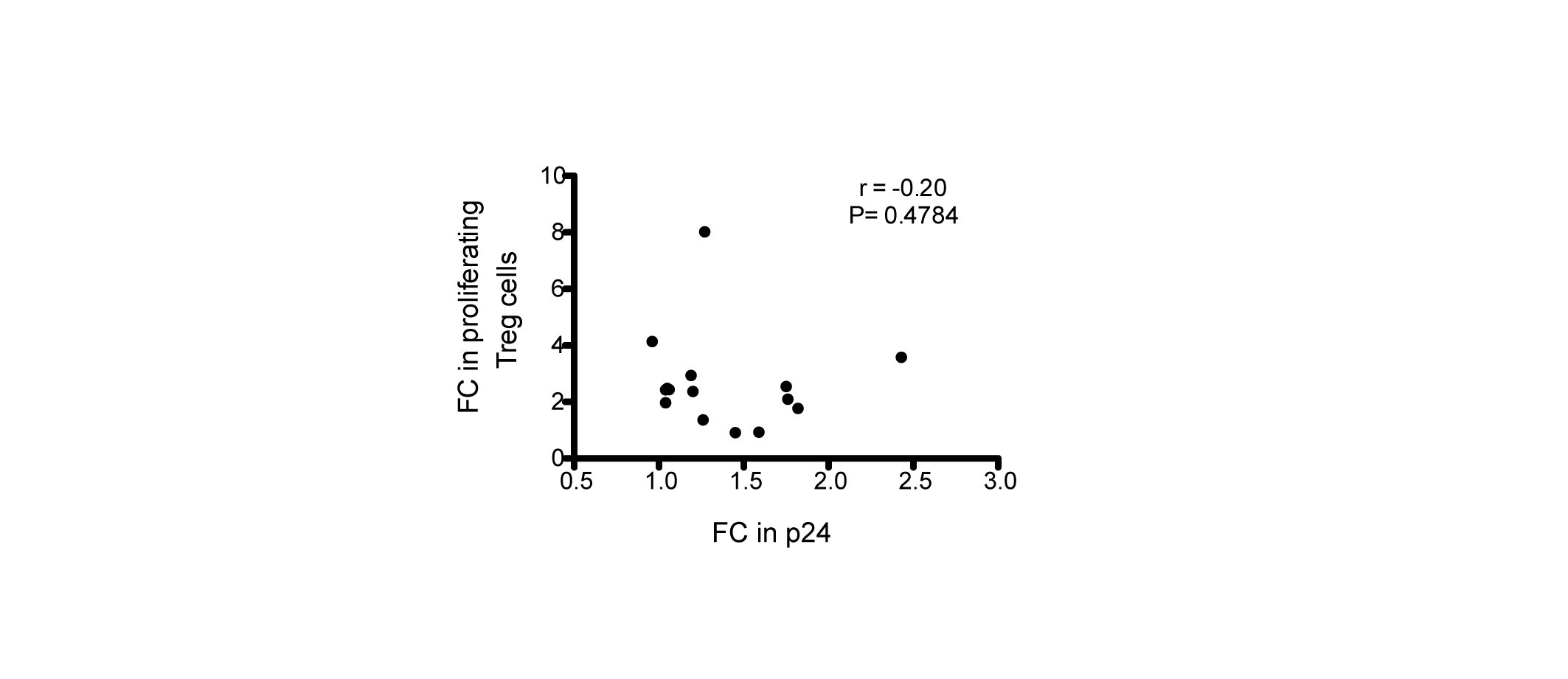

Supplement: S7 Fig — PBMCs were stimulated with Gag peptides in the presence of a PD-L1 blocking antibody or an isotype control antibody. After 4 days in culture, supernatants were harvest to quantify the p24 HIV core antigen by ELISA. Correlation between fold change in p24 and fold change in percentage of proliferating Treg cells cells is shown. Spearman’s rank correlation coefficient (r) and p value (P) are indicated. (TIF) [file ppat.1005270.s010.tif]
